# Data on GC/MS elution profile, ^1^H and ^13^C NMR spectra of 1-, 3-, and 6-Nitrobenzo[*a*]pyrenes

**DOI:** 10.1016/j.dib.2019.104995

**Published:** 2019-12-12

**Authors:** Kefa K. Onchoke

**Affiliations:** Department of Chemistry & Biochemistry, Stephen F. Austin State University, Box 13006 – SFA Station, Nacogdoches, TX, 75962-13006, USA

**Keywords:** Nitrated benzo[*a*]pyrenes, ^1^H and ^13^C NMR, J-coupling constants, Elution profile, GC/MS, 1-NBaP, 3-NBaP, 6-NBaP

## Abstract

The data presented in this article is related to the research article entitled, “^13^C NMR Chemical Shift Assignments of Nitrated Benzo[*a*]pyrenes based on Two-dimensional Techniques and DFT/GIAO Calculations”, Kefa K. Onchoke, J. Chem. Sci. (2020) [1]. The NMR spectral profiles of nitrated benzo[*a*]pyrenes is presented. Further, the article describes elution profiles of 1-, 3- and 6-NBaP, the acquisition of ^1^H and ^13^C NMR data and the J-Coupling constants (which are useful for the assignment of peaks via 2D HMQC and HMBC techniques). The data presented is useful for developing structure-activity relationships for other nitrated polycyclic aromatic hydrocarbons (NPAHs).

**Table:** Specifications Table

Subject area	*Environmental Chemistry*
More specific subject area	*Nitrated polycyclic aromatic compounds*
Type of data	*Table, graph, figure*
How data was acquired	*NMR spectroscopy. UV–Vis were used in the study. GC-MS chromatograph* (a) A Bruker 500 MHz NMR spectrometer was used for acquisition of ^1^H and^13^C NMR chemical shifts in ppm, downfield from internal tetramethylsilane (TMS). Samples were acquired in analytical grade CDCl_3_. (b) The UV–vis spectra of the NBaPs were in agreement with literature reports reported in Ref. [2]. (c) GC-MS Chromatograph: A Finnigan Ultra Trace GC-Mass DSQ spectrometer (or Electron impact mass, 70 eV MASPEC II system) in the positive ion chemical ionization mode was used. (d) Thin layer chromatography (TLC) was performed on precoated silica gel on alumina (Aldrich). Preparative TLC plates (from Analtech Inc., 20 × 20 cm, 1000 μm silica gel) were used for separating benzo[*a*]pyrene from the nitrated compounds (Fig. 1). Unless otherwise stated, 20% benzene in hexane was the TLC solvent system of choice. Both column chromatographic and Medium Pressure Liquid Chromatography MPLC separations were performed. MPLC separation was conducted on a Lobar Fertisgsante Gröβe β 310–25, LiChroprep® Si 60 of 40–63 μm particle size column from Merck. A Gilson automatic fractionator was used for collecting fractions.
Data format	Raw and analyzed
Experimental factors	(a) For NMR analysis: Benzo[*a*]pyrene was obtained from Sigma-Aldrich (Milwaukee, WI). The 1-, 3-, and 6-nitrobenzo[*a*]pyrene (1-, 3-, and 6-NBaP) were synthesized by known published procedures [[3], [4], [5]] and separated on a silica gel column (200–430 mesh) and washed with hexane (shown in Fig. 1). NMR analytical grade solvents were purchased from Aldrich Chemical Co. (Milwaukee,WI)
Experimental features	Nitrated PAHs are known carcinogens, mutagens and teratogens found in the environment. We provide the NMR spectral data necessary for characterization of the mononitrated BaP derivatives in the environment.
Data source location	The Ohio State University and Stephen F. Austin State University, USA
Data accessibility	All data are available within this article.
Related research article	Data in this article is associated with the paper: “^13^C NMR Chemical Shift Assignments of Nitrated Benzo[*a*]pyrenes based on two-dimensional Techniques and DFT/GIAO Calculations”, Kefa K. Onchoke, J. Chem. Sci. (2020) [1]

**Table:** 

**Value of the Data** • The data provides important information for identification of nitrated benzo[*a*]pyrenes (BaP) derivatives in environmental samples [6]. The nitrated benzo[*a*]pyrenes derivatives are ubiquitous in environmental matrices such as soils, wastewater samples, drinking water resources, and air particulates. • The chromatographic elution profiles are important for identification and distinguishing of nitrated BaPs. This data can also be used for rationalizing structure-mutagenicity relationships in toxicological studies. • The data serves as a benchmark for other researchers analyzing mononitrated benzo[*a*]pyrene isomers generated from high temperature emissions. • The^13^C NMR spectra is useful for referencing and identification of NPAHs prevalent in biosolids/wastewater sludge [7].

## 1 Data

The dataset contains raw TLC and GC elution profiles (Fig. 1, Fig. 2, Fig. 3) and the MS fragmentation patterns (Fig. 4) of benzo[*a*]pyrene and its mononitrated derivatives (BaP, 1-, 3-, and 6-NBaP, Scheme 1). In addition, the ^1^H and ^13^C NMR data of BaP, 1-, 3-, and 6-NBaP are presented in Fig. 5, Fig. 6, Fig. 7, Fig. 8, Fig. 9, Fig. 10, Fig. 11, respectively. The actual raw data files are included in this article.

**Fig. 1:**
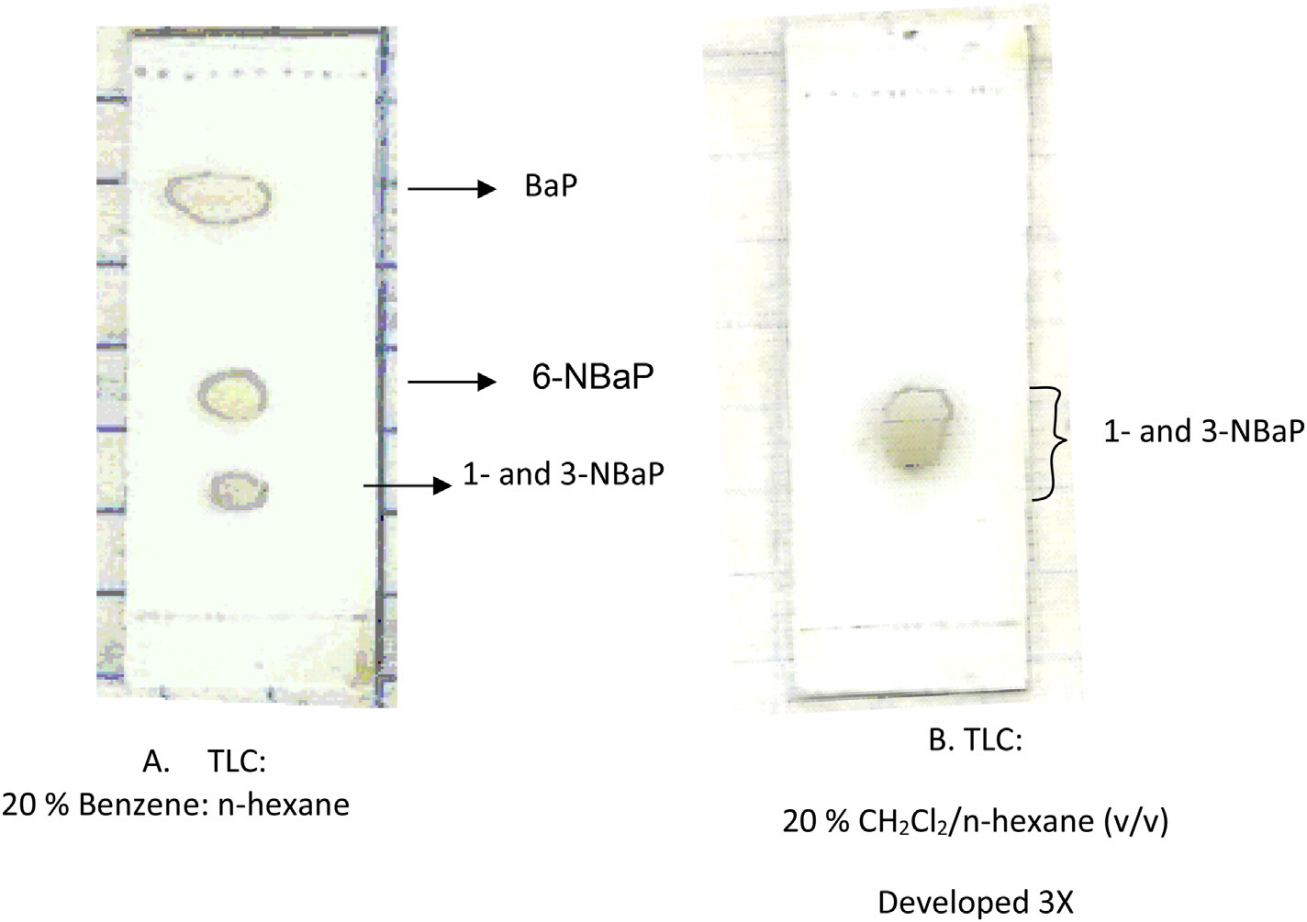
TLC of: (A) 1- and 3-NBaP, 6-NBaP and BaP developed 1*X* in 20%/hexane, (B) 1-, and 3-NBaP developed 3 times in 20% CH_2_Cl_2_/hexane (v/v) for the MPLC separation. R_fs_: (A) 0.619, 0.286, and 0.143 for BaP, 6-NBaP, and 1-and 3-NBaP respectively; (B) 0.317–0.439 for mixture of 1-, and 3-NBaP.

**Fig. 2:**
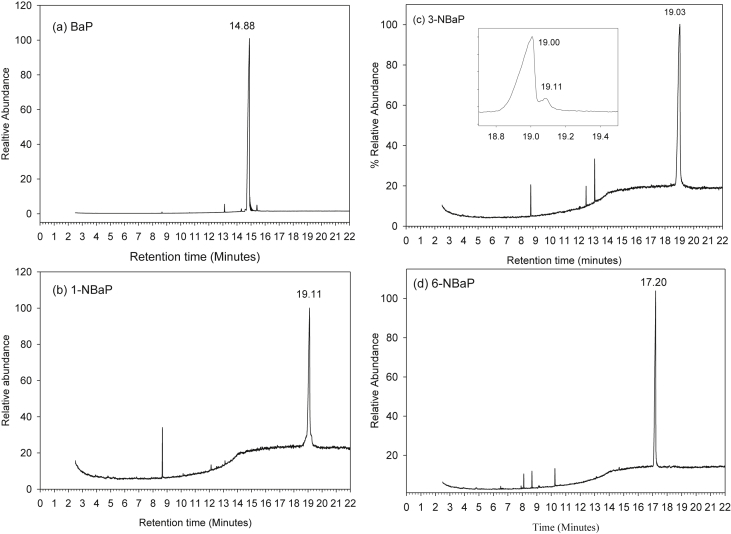
GC/MS profiles of; BaP, 1-NBaP, 3-NBaP, 6-NBaP, (a–d) respectively, separated on a 15 m 5% phenylmethyolysiloxane column. Inset in 2(c) is a chromatographic sample showing 1-NBaP at 19.11 minutes separated on a 15 m 5% phenylmethylysiloxane column.

**Fig. 3:**
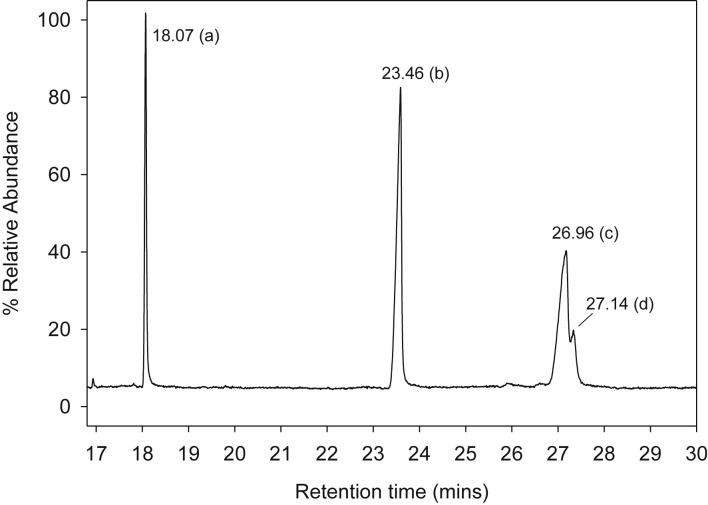
GC/MS profiles of a mixture of; (a) BaP, (b) 6-NBaP, (c) 3-NBaP, and (d) 1-NBaP separated on a 30 m 5% phenylmethylysiloxane (XTI-5) column.

**Fig. 4:**
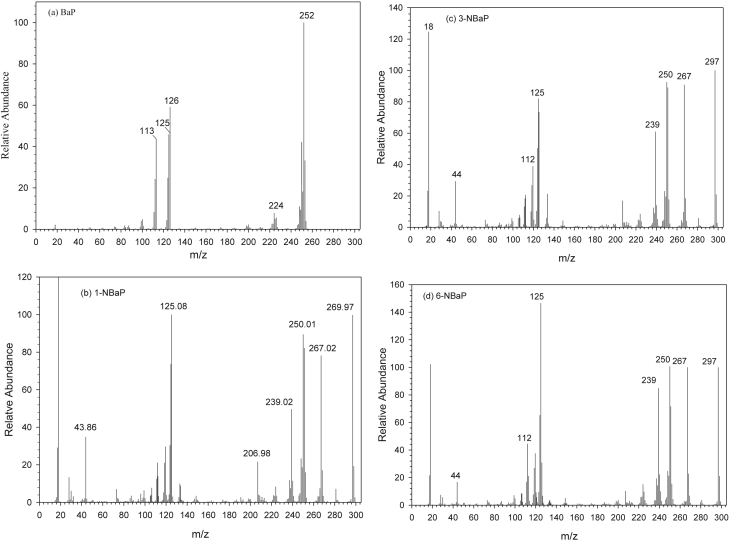
Electron Impact mass spectrum of the molecular ion peak of BaP, 1-NBaP, 3-NBaP, 6-NBaP (a-d, respectively).

**Scheme 1:**
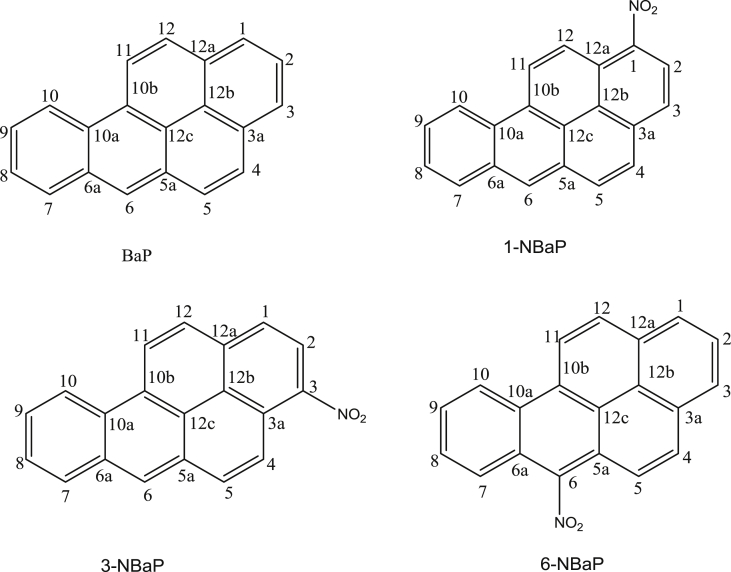
Numbering scheme and nitrated benzo[*a*]pyrenes (BaP), 1-, 3-, and 6-nitrobenzo[*a*]pyrenes (1-NBaP, 3-NBaP, and 6-NBaP).

**Fig. 5:**
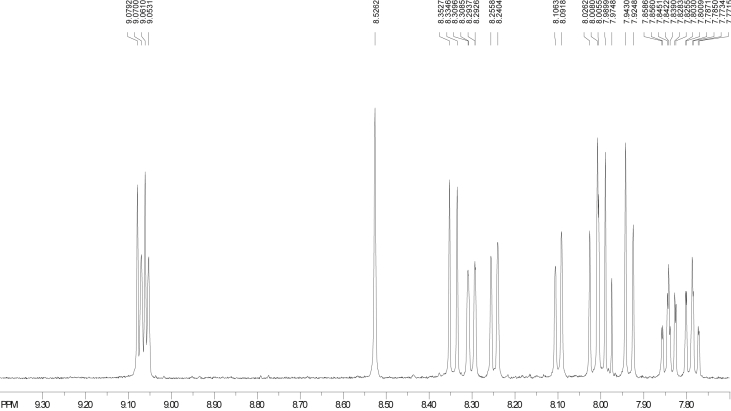
^1^H NMR spectra of benzo[*a*]pyrene (CDCl_3_, 500 MHz, Bruker).

**Fig. 6:**
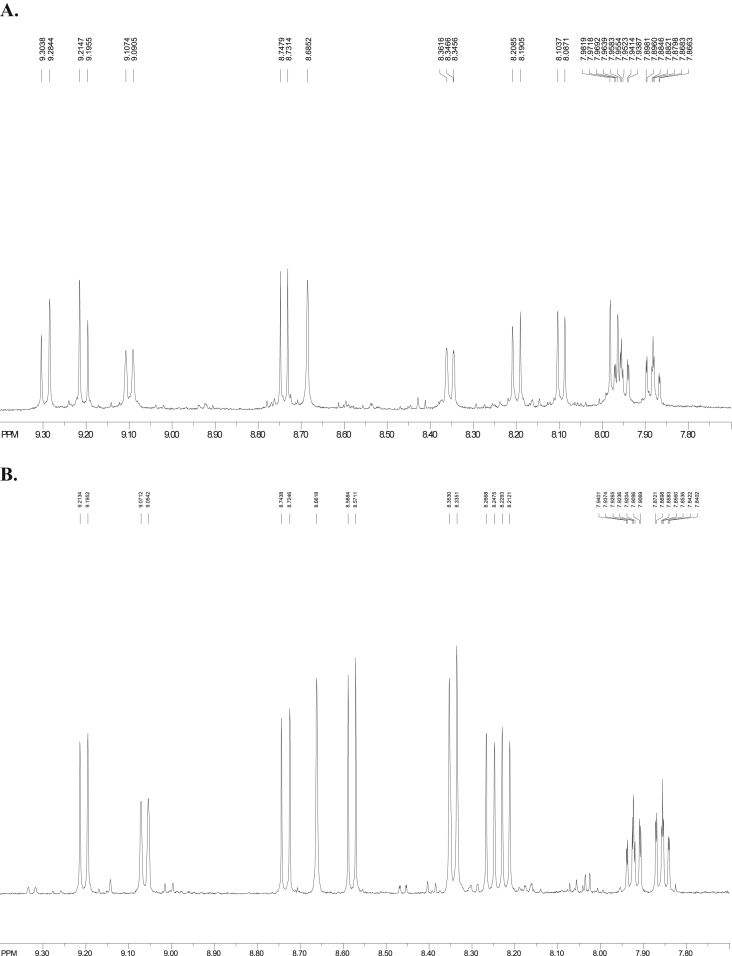
^1^H NMR of (A) 1-nitrobenzo[*a*]pyrene, (B) 3-nitrobenzo[*a*]pyrene (CDCl_3_, 500 MHz, Bruker).

**Fig. 7:**
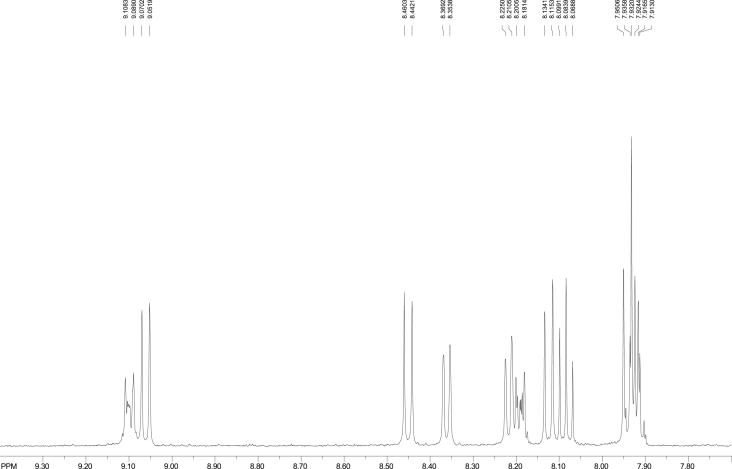
^1^H NMR of 6-nitrobenzo[*a*]pyrene (500.02 MHz, Bruker instrument, CDCl_3_, ppm).

**Fig. 8:**
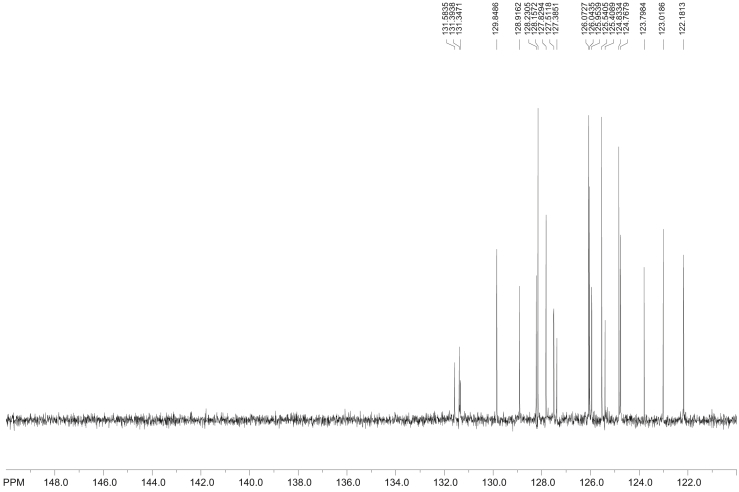
^13^C NMR spectra of benzo[*a*]pyrene (δ, CDCl_3_, 500 MHz, Bruker) transmitter frequency = 125.74 MHz.

**Fig. 9:**
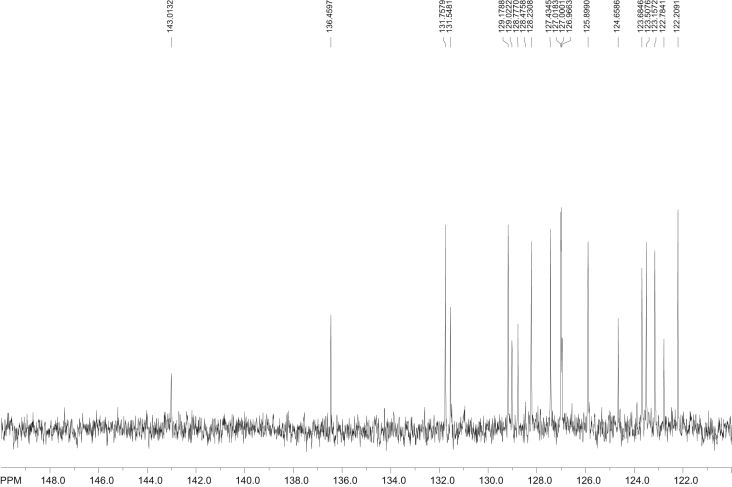
^13^C NMR spectra of 1-nitrobenzo[*a*]pyrene (1-NBaP, CDCl_3_, 500 MHz, Bruker, transmitter frequency = 125.74 MHz.), acquired in CDCl_3_.

**Fig. 10:**
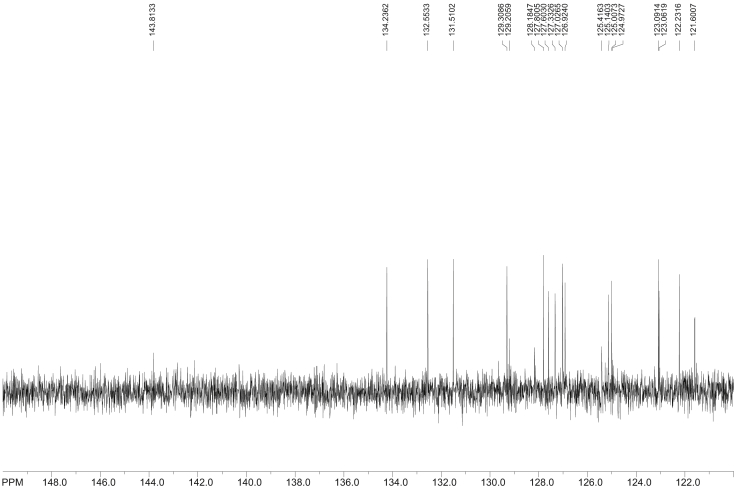
^13^C NMR spectra of 3-nitrobenzo[*a*]pyrene (1-NBaP, CDCl_3_, 500 MHz, Bruker), acquired in CDCl_3_. Transmitter frequency = 125.74 MHz.

**Fig. 11:**
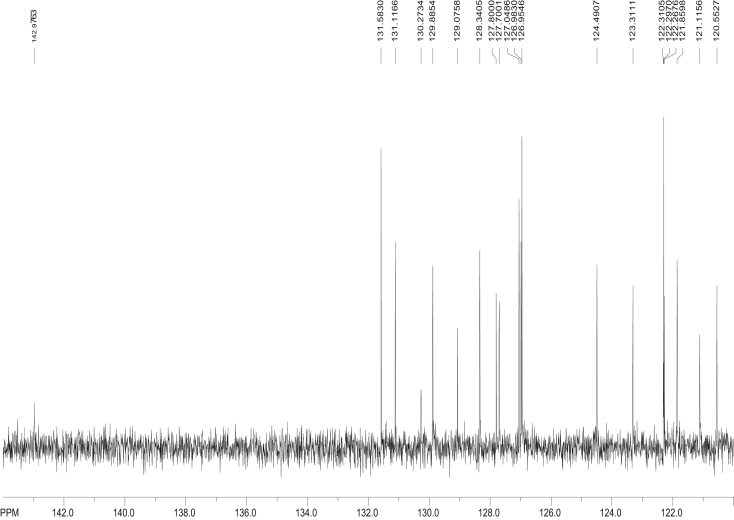
^13^C NMR spectra of 6-NBaP, Data acquired in CDCl_3_ (No calibration made, 10000 scans, Data presented are acquired).

The synthesis of 1-, 3-, and 6-NBaP and ^1^H acquisition is first presented prior to ^13^C NMR spectra (Fig. 5, Fig. 6, Fig. 7, Fig. 8, Fig. 9, Fig. 10, Fig. 11). Further information on the ^1^H chemical shifts and J-coupling constants of 1-, 3-, and 6-nitrobenzo[*a*]pyrene acquired in 500.1 MHz is presented in Table 1.

**Table 1:** ^1^H Chemical shifts and J-coupling constants of 1-, 3-, and 6-nitrobenzo[*a*]pyrene acquired in 500.1 MHz, d = doublet, s = singlet, t = triplet, m = multiplet.

Assigned designation/NitroPAH	BaP	1-Nitro-BaP	3-Nitro-BaP	6-Nitro-BaP
1	8.26–8.24 (d, J = 7.75)		8.57–8.59 (d, J = 8.56)	8.37–8.36 (d, J = 8.145)
2	8.01–7.97 (t, J = 15.55)	8.09–8.11 (d, J = 8.46)	8.20–8.22 (d, J = 8.62)	8.10–8.07 (t, J = 15.35)
3	8.11–8.09 (d, J = 7.32)	8.73–8.75 (d, J = 8.20)		8.23–8.21 (d, J = 7.27)
3a				
4	7.94–7.92 (d, J = 9.04)	7.96–7.98 (d, J = 9.00)	8.72–8.74 (d, J = 9.76)	7.95–7.93 (t, J = 9.42)
5	8.03–8.01 (d, J = 9.16)	8.19–8.21 (d, J = 9.13)	8.23–8.25 (d, J = 9.56)	8.14–8.12 (t, J = 9.64)
5a				
6	8.53 (s, J = 4263)	8.69 (s, J = 4342)	8.64 (s, J = 4322)	
6a				
7	8.31–8.19 (d, J = 8.32)	8.34–8.36 (d, J = 7.79)	8.32–8.36 (t, J = 9.09)	8.23–8.18 (m, d = 13.38)
8	7.80–7.77 (m, J = 15.92)	7.87–7.90 (m, J = 15.96)	7.85–7.88 (m, J = 15.93)	7.93–7.90 (m, J = 8.83)
9	7.86–7.83 (m, J = 16.76)	7.94–7.97 (m, J = 16.67)	7.92–7.95 (m, J = 16.57)	7.94–7.93 (m, J = 7.27)
10	9.08–9.07 (d, J = 4.68)	9.11–9.08 (d, J = 8.47)	9.05–9.06 (d, J = 8.53)	9.11–9.09 (m, J = 9.90)
10a				
10b				
11	9.06–9.05 (d, J = 4.02)	9.30–9.28 (d, J = 9.53)	9.20–9.17 (d, J = 8.98)	9.07–9.05 (d, J = 9.27)
12	8.35–8.33 (d, J = 9.35)	9.19–9.21 (d, J = 9.53)	8.32–8.36 (t, J = 7.62)	8.46–8.44 (d, J = 9.03)
12a				
12b				
12c				

## 2 Experimental design, materials, and methods

The experimental methods and procedures that allowed the data here presented are described in Ref. [1]. Here, only the protocol for NMR acquisition for ^1^H and ^13^C NMR are provided usually omitted in research articles due to the words limit.

### 2.1 GC/MS analysis

Benzo[*a*]pyrene and mononitro-BaPs were analyzed by Finnigan Ultra Trace GC-Mass DSQ spectrometer (or Electron impact mass, 70 ev MASPEC II system) in the positive ion chemical ionization mode. The analytical columns were 15-m and/or 30-m X 0.25-mm i. d. TR-5MS (Thermo Electron Corp., CA) and XTI-5 (0.25 mm id, 0.25 μm df, Reseek Corp., PA) fused silica capillary columns containing a 5% phenyl polysilylphenylenesiloxane phase with a 0.25 μm film thickness. The initial oven temperature was held at 40 °C for 2 min, then increased at 20 °C/min to 280 °C and held for 10 min. The carrier gas, helium, was held at a constant flow of 1 mL min^−1^. All injections were 1.0 μL on column. The transfer line was maintained at 300 °C. A splitless mode of effluent from the analytical column was let into the mass ion source.

For all GC/MS analyses, the total ion chromatograms (Fig. 2, Fig. 3) and selected ion monitoring (SIM) was used for assessing fragmentation patterns of the ions of interest (Fig. 4a–d). The molecular ion peak for BaP *m/z* 251 (M^+^) and nitro-BaPs *m/z* 297 (1-, 3-, and 6-NBaP, M^+^) (Fig. 4) were monitored.

### 2.2 NMR spectral data

#### 2.2.1 ^1^H NMR assignments

The ^1^H NMR spectra of BaP, 1-, 3- and 6-NBaP have been assigned previously in CDCl_3_ in acetone-d_6_ and DMSO [3,[8], [9], [10]]. Of relevance in this article are the ^13^C NMR peaks.

Fig. 5, Fig. 6, Fig. 7 depict the ^1^H NMR spectra of BaP, 1-, 3-, and 6-NBaP, respectively. The BaP singlet peak due to H-6 occurs at δ ≈ 8.53 ppm. The ^1^H resonance peaks due to H-6 were observed at δ values 8.69 and 8.64 p.m. for 1-, and 3-NBaP, respectively (Fig. 6A and B). In contrast, the characteristic ^1^H NMR spectra of 6-NBaP (shown in Fig. 7) lacks a singlet resonance peak at δ ≈ 8.53–9.00 ppm. The observed ^1^H absorption peak shifts are comparable to assignments by Johansen et al. [10]. Table 1 represents ^1^H chemical shifts and J-coupling constants of BaP, 1-, 3-, and 6-NBaP.

#### 2.2.2 ^1^H NMR spectra of 1-Nitrobenzo(*a*)pyrene (1-NBaP)

The ^1^H NMR spectra of 1-NBaP (500 MHz, CDCl_3_, Fig. 6A) spans 7.8–9.30 ppm and reveals groups of atoms. The singlet peak at 8.69 ppm is due to H-6 whilst the multiplet peaks at 7.97–7.94 (with a J-coupling constant of 16.67 Hz) and 7.90–7.87 ppm were clearly the peaks due to H8 and H9. At lower sample concentrations the H4 doublet peaks (8.01–7.99, 9.15 Hz) were resolved as triplets exhibiting long range interactions. At higher concentrations, the proton signals due to H4 overlap with those of H9 at 7.93–9.91 ppm. Because of the neighboring nitro group, the H12 is clearly deshielded to 9.22–9.20 ppm. The doublet peaks at 9.31–9.28 ppm and 9.11–9.09 ppm have previously been assigned to H11 and H10, respectively. By comparing the peak assignments with literature δ chemical shifts assigned as follows: H3 (d, 8.75–8.73, 8.20 Hz), H7 (d, 8.36–8.34 7.79 Hz), H5 (d, 8.21–8.19, 9.13 Hz), H2 (d, 8.11–8.09, 8.46 Hz) [11].

#### 2.2.3 ^1^H NMR spectra of 3-Nitrobenzo(*a*)pyrene

The ^1^H NMR spectrum (500.13 MHz, CDCl_3_, Fig. 6B) spans 7.85–9.19 ppm and reveals groups of atoms as follows. The doublet peaks at 9.19–9.18 and 9.06–9.05 ppm are assigned to H11 and H10, respectively. The singlet peak at 8.64 ppm must be due to H6 whilst the multiplet peaks at 7.95–7.92 and 7.88–7.85 ppm are due to H9 and H8, respectively. The proton spectrum at the H8 and H9 are clearly evident by the triplet splits. The proton signals due to H4, and H3 occur at 8.73–8.72 and 8.59–8.57 ppm, respectively. Because of the nitro group's electron withdrawing ability, the H12 is deshielded to 8.35–8.34 ppm. The rest of the peaks are assigned from and compared to literature [10] as follows: H1 (d, 8.59–8.57, 8.71 Hz), H4 (d, 8.74–8.72, 9.54 Hz), H7 (d, 8.34–8.32), H5 (d, 8.25–8.23, 9.58 Hz), H2 (d, 8.22–8.20, 8.62 Hz). The difference between 1-, and 3-NBaP, is particularly evident with H4, and the protons H2 and H5. 1-NBaP shows protons H2 and H5 occurring at a higher field compared to those in 3-NBaP. This is attributed to the extent of electronic interaction between the aromatic orbitals of the nitro group and those of the nitro group. The peri protons at H-2 and H-5 (in each 1-, and 3-NBaP) will greatly be shielded or deshielded.

#### 2.2.4 ^1^H NMR spectra of 6-Nitrobenzo(*a*)pyrene

The ^1^H NMR spectrum of 6-NBaP (depicted in Fig. 7) spans 7.90–9.11 ppm. Evidently, the absence of the singlet peak between 8.50 and 9.05 ppm, due to H6 in BaP, is strongly indicative of 6-NBaP. The multiplet peaks at 9.11–9.09 ppm due to H10 is indicative of an interaction of H10 with H9 and H11 and are the most shifted downfield. The doublet protons due to H11 are assigned to 9.05–9.07 ppm, whilst the multiplet peaks at 7.94–7.93 and 7.92–7.90 ppm are clearly the peaks due to H9 and H8, respectively. The proton signals due to H4 (*J* = 9.744 Hz) overlap with those of H9 and occur at 7.95–9.93 ppm. Because of the nitro group's electron withdrawing ability, the H12 is deshielded to 8.46–8.45 ppm. The rest of the peaks are assigned and compared to literature [10] as follows: H1 (d, 8.372–8.36, 7.527 Hz), H3 (d, 8.21–8.23, 7.265 Hz), H7 (m, 8.18–8.20, 9.67 Hz), H5 (d, 8.12–8.14, 9.32 Hz), H2 (t, 8.07–8.10, 15.124 Hz). The difference between 6-NBaP and 1-, and 3-NBaP is particularly evident at the H4, and the protons H2 and H5. The triplet chemical shifts due to H2 can easily be assigned vis-a-vis the H5 doublet peaks.

#### 2.2.15 ^13^C NMR assignments from HMQC/HMBC/Theory experiments

While one study reports ^13^C NMR assignments of 1-, and 3-NBaP in DMSO [9] none is available for 6-NBaP. The current study assigned ^13^C chemical shifts of three mononitrated benzo(*a*)pyrenes in CDCl_3_ via heteronuclear multiple quantum coherence (HMQC) and heteronuclear multiple-bond connectivity (HMBC) methods (ref # [11]). HMQC was used to assign chemical shifts based on direct C→H connectivity.

The proton-decoupled ^13^C NMR (with a Waltz decoupling routine, typically more than 10, 000 scans, 125.74 MHz, CDCl_3_) spectra corresponding to BaP, 1-, 3-, and 6-NBaP are shown in Fig. 8, Fig. 9, Fig. 10, Fig. 11, respectively. More than 2 mg of sample were dissolved in ∼1 mL of CDCl_3_ (99.85 atom % D). All spectra were recorded at 298 K in a 5 mm probe. In all cases HMBC experiments were optimized to detect aromatic couplings of ∼8 Hz. HMBC spectra were recorded with a 500.13 MHz spectrometer using states TPP1 to achieve phase sensitivity. Two hundred and fifty-six t_1_ experiments of 1280 real data points (120 scans) were recorded with a relaxation delay of 0.8 s.

The HMQC and HMBC spectra and δ chemical shift values of BaP, 1-, 3-, and 6-NBaP are the subject of the article in Ref. # 1 and are not discussed in this data report.
